# An Unusual Case of Hemothorax in an Ehlers-Danlos Syndrome Patient Following Sildenafil Use

**DOI:** 10.7759/cureus.55084

**Published:** 2024-02-27

**Authors:** Ashwin Varkey, Katherine Killian, Jillian Melnick

**Affiliations:** 1 Internal Medicine, Lenox Hill Hospital, New York, USA

**Keywords:** telemedicine, ehlers-danlos syndrome, hospital med, video-assisted thoracoscopic surgery (vats), spontaneous hemothorax

## Abstract

Ehlers-Danlos syndrome is a disorder of collagen production that affects the connective tissues of the body. It can cause several conditions and severely affect patients' quality of life and activities of daily living. Here, we present an unusual case of hemothorax in a patient with Ehlers-Danlos syndrome after sildenafil use. This manifested in shortness of breath and cough and prompted the patient to visit the emergency room. The hemothorax was treated surgically, and the patient recovered well. Sildenafil was initially prescribed via a telemedicine service without in-person consultation. It is important that physicans perform a thorough history and physical examination prior to prescribing medications to patients, especially those at risk for complications.

## Introduction

Ehlers-Danlos syndrome is a disorder of collagen production, which affects the connective tissues of the body. This condition is characterized by skin hyperextensibility, joint hypermobility, and easy bruising [1]. Here, we describe the case of a male in his mid-40s with Ehlers-Danlos syndrome who developed recurrent hemothorax after being prescribed sildenafil by an online prescription service. Online prescription services are becoming more prevalent in the United States and may impact medical care. Our patient used an online prescription service to obtain a medication for erectile dysfunction known as sildenafil and suffered a hemothorax shortly after taking it.

 In recent years, online prescription services have launched a format of telemedicine, which allows patients to obtain prescriptions for medication without interacting with a provider. In many instances, the patient fills out a series of questions that are reviewed by a medical provider, who then decides whether to proceed with the prescription [2]. In other instances, the patient communicates with a medical provider via a messenger application. In both scenarios, the patient is not physically seen by a provider, nor do they speak via telemedicine devices. This mechanism bypasses the ability to perform a physical exam.

In this case, we present a patient who used an online prescription service to obtain a medication for erectile dysfunction known as sildenafil, a phosphodiesterase five (PDE-5) inhibitor. PDE-5 inhibitors have been associated with intraluminal tears and have been shown to have an association with aortic dissection in men [3]. A 2012 case report described a 63-year-old male with hypertension who presented to the emergency room with chest pain 20 hours after taking tadalafil, a PDE-5 inhibitor, and was found to have a type B dissection of his descending thoracic aorta [4]. In addition to this case, a 61-year-old male with hypertension and atrial fibrillation was found to have a type A aortic dissection 30 minutes after ingesting sildenafil [5]. Type B aortic dissection has also been reported after ingestion of cocaine and sildenafil [6].

Based on these prior reports and the current case, there has been an association seen between phosphodiesterase inhibitors and vessel injury. A common feature of these cases is a predisposition to vessel fragility. In the case of our patient, this was his underlying Ehlers-Danlos system, and in the other cases, it was hypertension and cocaine use, all of which may predispose blood vessels to tears. There is a scarcity of data surrounding adverse reactions of phosphodiesterase inhibitors in individuals with Ehlers-Danlos syndrome. Online prescription services lack thorough history taking and physical exams that may have contributed to this patient suffering a hemothorax as a complication of sildenafil. A hemothorax is characterized by the accumulation of blood in the pleural cavity, which is the space between the chest and the lung wall. This condition occurs following damage to blood vessels in the chest, which most often is due to trauma but can also be due to medical conditions affecting blood clotting or the integrity of blood vessels [7]. Here, we present an unusual case of hemothorax caused by sildenafil in a patient with Ehlers-Danlos syndrome.

## Case presentation

A male in his mid-40s with Ehlers-Danlos syndrome complicated by prior bilateral carotid dissection, bilateral iliac artery dissections, and spontaneous pneumothorax was admitted to the hospital for acute onset chest pain after sexual intercourse. At that time, a computed tomography (CT) scan of the chest, abdomen, and pelvis revealed a moderate left-sided pleural effusion with concern for hemothorax. A left-sided pigtail catheter was placed, resulting in the diagnosis of a hemothorax. The chest tube was subsequently removed, and the patient was discharged home. Further information surrounding outside hospitalization was unable to be ascertained.

The patient prior to admission was taking atorvastatin 80 mg once daily and pantoprazole 40 mg once daily and at the time was not taking any non-steroidal anti-inflammatory drugs, antiplatelet drugs, selective serotonin reuptake inhibitors, or selective norepinephrine reuptake inhibitors. The patient did report prior to the chest pain that he had taken one 40 mg dose of sildenafil before sexual intercourse. The information surrounding his home medications was not acquired until he followed up with his primary care physician following hospitalization. The patient was unable to specify the specific timing of the sildenafil use. He reported he had not previously used sildenafil. The patient had a family history significant for Ehlers-Danlos syndrome. His social history was significant for social alcohol use and no tobacco or recreational drug use. He drank about one to two alcoholic mixed drinks per month. He was sexually active and worked in construction.

In the weeks following hospital discharge, the patient developed a progressive cough and left chest wall pain. He visited his primary care provider two weeks following discharge, the information surrounding taking sildenafil was ascertained by his primary care physician at his visit, and he was advised to stop taking any further sildenafil doses. The patient reported that he had taken additional sildenafil doses, but he was unable to quantify how many doses. He reported that he had been receiving sildenafil prescription from a telemedicine service. A chest X-ray was ordered by his outpatient primary care provider, which showed a recurrent left-sided pleural effusion. The patient was advised to return to the emergency room one day after chest X-ray by his primary care physician.

**Figure 1:**
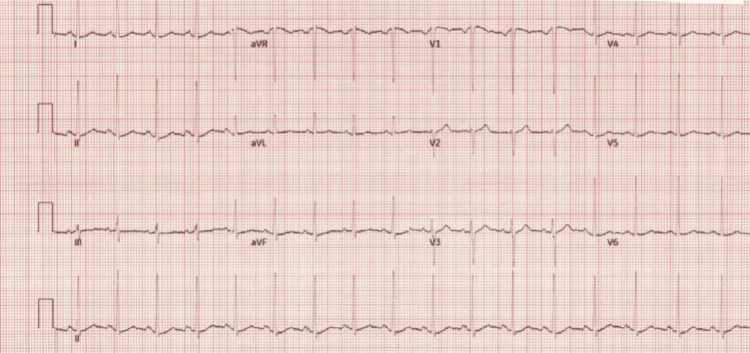
EKG revealing sinus tachycardia of the patient upon admission

**Figure 2:**
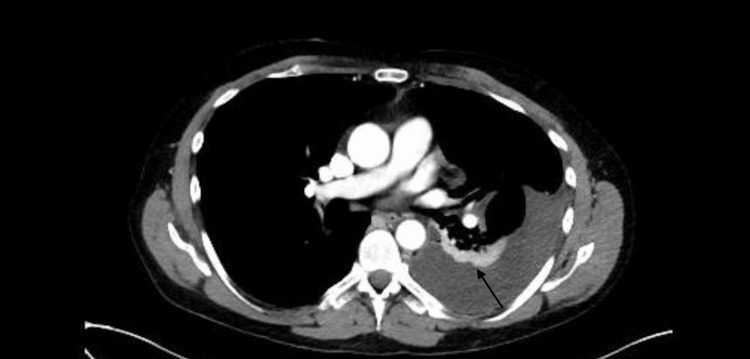
Chest computed tomography demonstrating a large left pleural effusion The arrow indicates a hematocrit sign. There are two different densities in the collection because cellular components in effusion settle to a dependant region. The Hounsfield units were compatible with subacute blood products. The Hounsfield units were 49 HU.

In the emergency department, his vitals were as follows: temperature 99.6 F, heart rate 117, blood pressure 120/66, respiratory rate 18, and oxygen saturation 98% on room air. A review of systems was notable for cough and left-sided chest wall pain. Physical examination exhibited decreased lung sounds at the base of the left lung but was otherwise unremarkable. Labs revealed normocytic anemia with a hemoglobin of 10.4 g/dL(normal range for males, 13.0-17.0 g/dL). The remainder of the complete blood count was normal, there was no leukocytosis, and troponin was within normal limits. An electrocardiogram showed sinus tachycardia with left ventricular hypertrophy; no ST-T changes were present (Figure 1). Chest computed tomography demonstrated a large left pleural effusion with predominantly low-density simple fluid compatible with evolving subacute versus chronic blood products (Figure 2), as well as small residual hyperattenuating blood products at the level of the posterior left lung base.

No pulmonary embolism or pneumothorax was present.  The large left pleural effusion noted on CT imaging of the lung was graded as most compatible with subacute/chronic hemothorax with variable age blood products. CT is considered the gold standard for diagnosis of hemothorax [8]. To further elucidate the diagnosis and perform evacuation of hemothorax, the patient had left video-assisted thoracoscopic surgery (VATS) from which fluid analysis and pleural biopsy were performed. The patient’s pleural fluid culture grew no organisms, and pathology revealed mesothelial cells, macrophages, small lymphocytes, few acute inflammatory cells, and abundant red blood cells. The abundant red blood cells and acute inflammatory products further strengthened the diagnosis of hemothorax. The diagnosis of hemothorax was confirmed with CT imaging in conjunction with pathology and the absence of growth on the pleural fluid culture.

**Figure 3:**
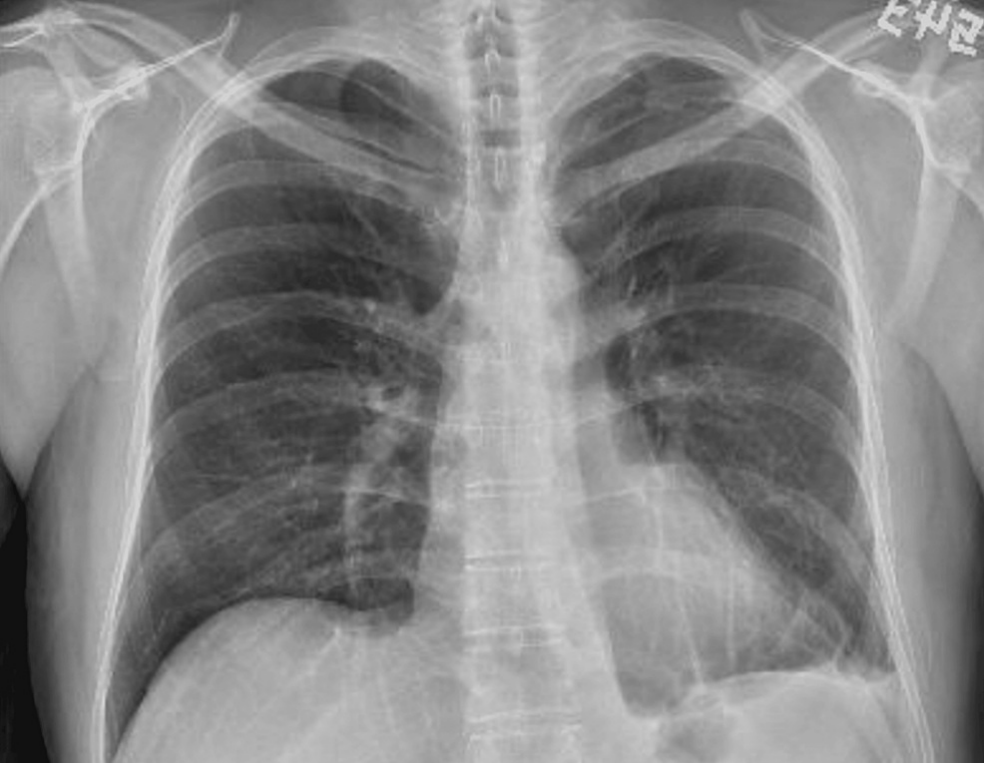
Follow-up chest X-ray with resolution of left-sided hemothorax.

The patient was admitted to the cardiothoracic surgery service. The patient left the operating room with three chest tubes, all of which were removed on postoperative days 3 and 4. The patient was discharged on postoperative day 4.   At a primary care follow-up appointment six weeks after admission, the patient reported feeling well, with no acute complaints. His cough and chest pain had resolved, and he had no shortness of breath. He followed up with his cardiothoracic surgery team six weeks after discharge, and his repeat chest X-ray at that time was negative for recurrent pleural effusion or hemothorax (Figure 3).

He has not taken any further sildenafil doses. The patient was able to return to work with no complications and resume his normal lifestyle with consistent follow-up with his primary care physician and vascular surgeon. He does not need to follow up consistently with his cardiothoracic surgeon.

## Discussion

Online prescription services are often seen as a convenient and affordable option for obtaining prescription drugs, but this system does have limitations. There are currently three types of online prescription services. The first category consists of online pharmacies that function to provide medication once a prescription from a physician is submitted. The second category includes websites that employ doctors and pharmacists and involve a questionnaire or video conference between the physician and patient. The third category is referred to as an online drug shop, which differs from type 2 in the way that a questionnaire or video is not necessary to obtain medication. In type 3, consumers simply pick out the medication and pay with a credit card, which is considered illegal by most law enforcement agencies [9]. 

In the case of our patient, he obtained a medication known as sildenafil, from the second category of online prescription services described above. There is a previously cited case of a 52-year-old male who died of a heart attack after taking sildenafil that he bought from an online source that only required a questionnaire to obtain the medication. FDA officials who commented on this case reported that although there is no proof linking this death to the drug, they believe a traditional doctor-patient relationship may have prevented this [10]. There are prior studies in which researchers investigated whether drugs are supplied to people for whom they are not suitable, in this scenario, sildenafil. To assess this, they posed as patients with obvious contraindications to this medication, such as coronary artery disease, or currently taking a nitrate. Despite these contraindications, 20-30% of patients received sildenafil [11]. Advantages of traditional doctor-patient interactions over online prescribing include discussion of patients' past medical history, current health problems, and physical examination. 

In the case of our patient, he was prescribed a PDE-5 inhibitor, sildenafil, via an online prescription service despite his risk of vascular complications.  The direct link between PDE-5 inhibitors and hemothorax is not firmly established, although there are numerous case reports demonstrating an association between PDE-5 inhibitors and vascular smooth muscle cells. To further discuss association, it is important to understand the physiology of PDE-5 inhibitors. PDE-5 is the predominant phosphodiesterase in vascular smooth muscle cells that hydrolyzes cGMP. By inhibiting this enzyme, there is the prevention of the degradation of cGMP, which activates kinase G, leading to the relaxation of vascular smooth cells and ultimately dilation of blood vessels [12]. It is likely that patients with risk factors affecting vasculature (such as a history of aneurysms, hypertension, cocaine abuse, or genetic conditions, such as our patient with Ehlers-Danlos syndrome) are at a higher risk of detrimental complications. Upon a review of the literature, we found similar cases, one with a post-sildenafil type A aortic dissection and another with a type B dissection after the use of cocaine and sildenafil [13]. In addition, there have been associations seen in mice who have had pre-existing abdominal aortic aneurysms worsen after the use of PDE-5 inhibitors [14]. It has been hypothesized that sildenafil and possibly other PDE-5 inhibitors have the potential to increase blood pressure in situations, such as sexual activity, which can provoke aortic dissection [15]. Despite these case reports, there are still no data promoting direct evidence of hemothorax caused by sildenafil.

A similarity between these cases and ours is the fact there was no mechanical stress of trauma, but there were various predisposing factors, such as known aneurysms. An additional case report reflects a type B aortic dissection after the use of tadalafil [5]. Although a different PDE-5 inhibitor, this patient was more like ours in that he took said medication once, rather than chronically in the other case reports [15]. The vascular risks of PDE-5 inhibitors are not entirely understood, but the evidence above suggests an underlying association that may be affected by various risk factors and chronicity of use. In the changing world, it is important for practitioners and patients to adapt. Some states, such as Washington, have adopted telemedicine guidelines addressing this issue. Patient completion of a questionnaire does not, by itself, establish a practitioner-patient relationship, and therefore treatment, including prescriptions, based solely on a questionnaire does not constitute an acceptable standard of care [10].

## Conclusions

It is imperative to establish a doctor-patient relationship with a thorough history and physical examination prior to prescribing new medications to patients. This case illustrated the adverse side effects of a patient with Ehlers-Danlos syndrome taking a phosphodiesterase inhibitor. Connective tissue diseases and vascular diseases have been shown as a population at risk for vessel fragility and adverse outcomes. Phosphodiesterase inhibitors should be prescribed cautiously in patients with connective tissue disorders. Telemedicine has an important role in our changing clinical world, but it must be used cautiously and not as a sole replacement for in-patient visits. This tenant is even more important in cases involving patients with extensive medical problems.
